# Encouraging Realistic Expectations in STEM Students: Paradoxical Effects of a Motivational Intervention

**DOI:** 10.3389/fpsyg.2016.01109

**Published:** 2016-07-26

**Authors:** Nathan C. Hall, Anna Sverdlik

**Affiliations:** Department of Educational and Counselling Psychology, McGill UniversityMontreal, QC, Canada

**Keywords:** motivational intervention, STEM, overconfidence, downgrading expectations, higher education

## Abstract

College students in STEM (science, technology, engineering, mathematics) disciplines are increasingly faced with highly competitive and demanding degree programs and are at risk of academic overconfidence. Following from theory and research highlighting the psychological and developmental risks of unrealistic expectations, the present exploratory study evaluated the longitudinal effects of a motivational intervention encouraging college students in STEM degree programs (*N* = 52) to consider the importance of downgrading one’s expectations in response to academic setbacks. Contrary to study hypotheses, the results showed intervention participants to report significantly higher expectations and optimism on post-test measures administered 4 months later, no significant gains in emotional well-being or achievement goal orientations, and lower GPAs over five subsequent semesters. These paradoxical effects underscore the need for additional larger-scale research on the nature of students’ responses to potentially ego-threatening motivational programs in STEM disciplines so as to minimize achievement deficits at the expense of preserving motivational resources.

## Introduction

For many students, adaptation to college life includes both academic and developmental demands, with students in demanding degree programs facing numerous challenges including marked pressure to succeed academically, increased expectations for independence and maturity, as well as the need for successful adjustment to unfamiliar tasks and environments (Bozick, 2007; Schrader and Brown, 2008). These and other transition-related demands can serve to impede student success, with many bright and motivated students experiencing academic setbacks due to their inability to successfully adapt – a “paradox of failure” that for many leads to disengagement and dropping out (Perry et al., 2005). This paradox is especially prevalent among STEM (science, technology, engineering, mathematics) students, as failure in these highly demanding degree programs is very costly not only to one’s motivation but career potential (Rask, 2010). In response to these academic realities, students in STEM programs are often found to exhibit academic overconfidence in an effort to reconcile their high prior achievement with the highly aversive nature of potential failure (Armor and Sackett, 2006; Reuben et al., 2013) which can further contribute to academic disengagement after setbacks (Perez, 2012). In an effort to redress this problematic trend, the present study evaluated the long-term effectiveness of a motivational program encouraging realistic aspirations for academic success among STEM undergraduates so as to improve psychological adjustment and preserve long-term achievement outcomes for students in these challenging academic domains.

## Challenges in STEM Disciplines

For most undergraduates, the selection of one’s major is reflective of their identity and critical to their psychological well-being throughout their studies and future career (Galotti, 1999). For students in STEM disciplines, this decision is often accompanied by significant anxiety and impairments to physiological and psychological health due to heightened competition and the resulting need for superior academic performance (Wai et al., 2010). For these reasons, STEM students, particularly females, are more likely to switch to a non-science major, effectively opting out of a natural science career (Daempfle, 2003; Rask, 2010; Lee, 2011), prompting institutional as well as national efforts to retain students in STEM disciplines (Perez, 2012). Not surprisingly, two of the most often cited reasons for opting out of STEM disciplines include loss of interest (Seymour and Hewitt, 1997) and academic difficulties in STEM courses with respect to both absolute GPA and one’s grades relative to peers (Strenta et al., 1994; Rask, 2010; for more on competition and the Big-Fish-Little-Pond effect among STEM undergraduates, see Almarode et al., 2014; Van Soom and Donche, 2014). Accordingly, students identified as having high ability in science disciplines are also likely to leave STEM majors due to considerable academic pressure and associated physical and psychological distress (Webb et al., 2002).

As such, recent research underscores the need for greater research on motivational variables in STEM students related to interest, perceived ability, and expected success that represent important predictors of engagement, achievement, and attrition in the natural sciences (Perez, 2012). More specifically, given the particular threats to physical and psychological health for students in competitive STEM programs, it is critical that students’ perceptions of their personal resources and academic expectations be adaptive in accurately reflecting the challenging realities of their chosen post-secondary domain (Moore and Healy, 2008). Appropriately calibrated expectations are of particular significance for students in demanding degree programs with pursuing unattainable goals having been found to result in student disengagement (Wrosch et al., 2003), performance declines (Rask, 2010; Perez, 2012), and impaired psychological well-being (Seymour and Hewitt, 1997; Wrosch et al., 2003) due to high personal investment (e.g., time, effort, expenses, deferred personal goals). It is therefore hypothesized that an inability to accurately assess one’s potential success, more specifically, an overestimation of one’s personal resources, can lead to unrealistic academics expectations for students in demanding STEM programs that, if not met, could serve to erode subsequent motivation, performance, and well-being (Armor and Sackett, 2006).

## Academic Overconfidence

Overconfidence in higher education has consistently been found to adversely affect students’ personal and academic development. Although moderate optimism has consistently been demonstrated to be beneficial for maintaining achievement motivation (Krypel and Henderson-King, 2010), overly optimistic expectations concerning one’s academic performance has also been observed to have negative consequences in achievement settings. For example, Stone (2000) suggests that academic overconfidence can impair self-regulated learning due to inaccurately calibrated perceptions of knowledge gains (i.e., overestimating how much one has learned) and, consequently, neglecting to develop more advanced self-regulation skills (e.g., self-monitoring, reflection, realistic goal-setting) that are crucial for academic success. Additionally, numerous studies suggest that although overoptimistic expectations may be adaptive in the short term for motivation and self-esteem, they can also be detrimental for students’ long-term goal attainment (Robins and Beer, 2001; Klein and Helweg-Larsen, 2002; Nowell and Alston, 2007).

In two studies with undergraduates, Robins and Beer (2001) found that students who were particularly invested in a learning task (i.e., high ego involvement) were more likely to inflate their self-perceptions, report greater narcissism, and inaccurately evaluate their personal performance, suggesting that self-enhancement in challenging achievement settings may be used as a defensive strategy to maintain self-esteem (Lobel and Teiber, 1994). Further, these authors found self-enhancing students to disengage from their studies sooner over a 4-year period, as evidenced by lower self-esteem and graduation rates. These findings are consistent with those of a meta-analytic review by Klein and Helweg-Larsen (2002) who found academic overconfidence (optimistic bias) to correspond with a lower perceived risk of academic disappointment relative to one’s peers (cf. miscalibrated perceptions of academic ability; Kruger and Dunning, 1999) as well as longitudinal research from a developmental perspective (Heckhausen and Schulz, 1995) showing overconfident undergraduates to neglect cognitive strategies for maintaining persistence and performance after initial setbacks (e.g., positive reappraisal; Hall et al., 2006b).

It is important to note that greater optimism is typically beneficial for students, leading to lower stress, lower devaluing of educational goals, and more adaptive academic coping strategies as compared to pessimistic beliefs (e.g., Krypel and Henderson-King, 2010). However, social-psychological research in higher education also shows overly high optimism levels to predict a tendency to deny information that threatens one’s self-worth; a defensive strategy corresponding to both short-term emotional well-being and long-term educational deficits including inflated expectations and poorer levels of metacognition and achievement (Burson et al., 2006; cf. the “above average effect,” Kruger and Dunning, 1999). Findings further suggest that inaccurate perceptions of competence and future success are more likely in performance-oriented settings where information concerning contributing factors beyond ability is limited (Moore and Healy, 2008) and in demanding academic domains in which the likelihood of success is often uncertain (Burson et al., 2006).

With respect to studies addressing overconfidence specifically among students in STEM domains, emerging findings suggest that overconfident students are significantly more common in natural science disciplines as compared to business or humanities programs (Reuben et al., 2013; see also Schulz and Thöni, 2014). Findings further underscore the importance of confidence in one’s mathematical skills, over and above demonstrated ability levels, in predicting pursuit of STEM careers (Halpern et al., 2007) with perceptions of fixed high abilities corresponding to lower persistence, poorer learning strategies, and performance deficits in STEM courses (e.g., chemistry, pre-medicine students; Dweck, 2006). Males are also consistently found to overestimate their math abilities relative to females (e.g., Goetz et al., 2013), with recent finding showing gendered confidence beliefs to predict stronger intentions to pursue math-intensive post-secondary studies (e.g., Sáinz and Eccles, 2012; Bench et al., 2015; Simon et al., 2015). This pattern of overconfidence in STEM, as typically represented by higher performance expectations among males relative to females in mathematics and science domains despite equivalent aptitude (e.g., Hyde et al., 2008), as well as corresponding gender stereotypes (e.g., Brotman and Moore, 2008), are often cited as contributing to disproportionately lower enrollment and greater dropout among women in STEM domains (e.g., Larose et al., 2006; Ceci and Williams, 2010; Cheryan, 2012).

## The Motivational Theory of Life-Span Development

As noted above, academic overconfidence has recently been examined from a developmental self-regulation perspective, with students adopting unrealistic motivational beliefs (e.g., high persistence, few back-up strategies) showing poorer psychological and achievement outcomes relative to their peers (e.g., Hall et al., 2006c). Following from the motivational theory of life-span development by Heckhausen et al. (2010), this work is based on premise that how individuals choose to interact with their environment depends largely on how much control they perceive over it. Individuals who perceive themselves as having personal control over changes in their environment tend to use motivational strategies aimed at improving the situation and modify their behavior to achieve their goals. These types of control behaviors and motivational strategies (e.g., persistence, seeking assistance) are referred to as primary control. For example, after a non-satisfactory grade on an exam, a student who believes that they can improve their performance is likely to invest more time studying to improve their grade.

On the other hand, Heckhausen and Schulz (1998, p. 53) postulate that “repeated experiences of failure may… lead to low perceptions of personal control” that pose “major threats to the individual’s motivational resources for future action.” Thus, when faced with critical setbacks, optimal adjustment is hypothesized to be better promoted by changing one’s cognitions through secondary control so as to reconcile the discrepancy between the environment and one’s expectations (see also Morling and Evered, 2006; Skinner, 2007). For example, after a non-satisfactory grade on an important exam, a student who attributes their performance to factors beyond their personal control (e.g., extreme test difficulty) may attempt to find the “silver lining” or downwardly adjust their expectations for future exams to compensate for the motivationally threatening nature of the experience. This adaptive use of secondary control strategies to compensate for past or potential failure experiences is further hypothesized to maximize future primary control efforts and personal development in critical life domains (e.g., work, health) across the life-span (Heckhausen et al., 2010).

Given the importance of selecting appropriately challenging goals during critical developmental periods (e.g., emerging adulthood), empirical research based on Heckhausen et al.’s (2010) theory has examined the role of compensatory secondary control strategies involving both positive reappraisal (e.g., benefit-finding) and downgrading (e.g., importance, aspirations) in educational settings. With respect to positive reappraisal, studies show that beyond the detrimental effects of maintaining high primary control in the absence of secondary control for overconfidence and achievement (e.g., Hall et al., 2006c), post-secondary students who calibrate their emphasis on persistence vs. positive reappraisal based on their grades are more motivated later in the academic year (Hall, 2008), with positive reappraisal further predicting better health outcomes (Hall et al., 2006a) as well as higher academic motivation and achievement levels (Hall et al., 2006c).

Concerning downgrading of unrealistic aspirations as a motivational strategy, although primary control (persistence) has consistently been found to predict career goal attainment for graduating high-school students (e.g., females; Haase et al., 2008), students are also typically found to downgrade their career aspirations in the months prior to graduation (e.g., calibrating one’s “dream job” based on employment opportunities; Heckhausen and Tomasik, 2002). Additionally, studies show downgrading aspirations after failure in finding employment after graduation to predict greater well-being compared to sustained primary control (Tomasik et al., 2009), a finding also observed following unsuccessful college applications (Tomasik and Salmela-Aro, 2012). Multiple studies further show undergraduates who shift their focus from unachievable to more attainable goals to report better well-being (Wrosch et al., 2003, 2007). In sum, research based on Heckhausen et al.’s (2010) theory consistently shows compensatory strategies involving positive reappraisal and downgrading to better predict goal attainment and well-being in challenging academic conditions than unmitigated persistence, suggesting that motivational programs in which these strategies are encouraged may be of benefit, particularly in disciplines characterized by academic overconfidence.

## Motivational Interventions and Academic Overconfidence

Existing motivation research has evaluated the benefits of intervention programs for struggling college students based on varied social-psychological perspectives including self-theories of intelligence (e.g., Aronson et al., 2002), expectancy-value theory (e.g., Durik and Harackiewicz, 2007; Acee and Weinstein, 2010; Hulleman et al., 2010; Shechter et al., 2011), and achievement goals (e.g., Barron and Harackiewicz, 2001; Hoyert and O’Dell, 2006), with intervention studies in STEM domains showing considerable promise (e.g., Betz and Schifano, 2000; Harackiewicz et al., 2012). However, the most extensive research on motivational interventions in higher education has to date been based on Weiner’s (2010) attribution theory. Referred to as “attributional retraining” (AR), these programs encourage college students to adopt personally controllable explanations for academic setbacks (primary control) with studies over the past 30 years showing motivational, emotional, and achievement benefits for primarily social science students (e.g., Forsterling and Morgenstern, 2002; Wilson et al., 2002; Haynes et al., 2009).

With respect to intervention content, AR programs are typically informational (vs. persuasive) in nature and highlight the motivational and achievement benefits of adopting attributions for disappointing academic experiences (e.g., low grades) that are personally controllable (e.g., lack of effort) as opposed to uncontrollable (e.g., low ability). Whereas the intervention typically results in modest yet consistent gains in motivation (e.g., mastery goals; Haynes et al., 2008) and achievement (e.g., GPA), findings consistently show the most substantial performance benefits for students at risk of poor performance due to initial low grades (e.g., Perry et al., 2010), maladaptive attributions (e.g., Struthers and Perry, 1996; Jackson et al., 2009), poor learning strategies (Hall et al., 2004, 2007), and low self-esteem (e.g., Hall et al., 2011). Additionally, multiple studies have recently demonstrated long-term psychological and achievement benefits of this program for overly confident post-secondary students.

In a study by Ruthig et al. (2004), AR led to better GPAs, test anxiety, and course attrition specifically for overly optimistic social science students relative to their peers. These findings were replicated by Haynes et al. (2006), showing AR to increase attributions to effort specifically among overly optimistic social science students as well as improve both course-specific and cumulative achievement outcomes. Finally, an intervention study by Hall et al. (2006b) incorporated both Weiner’s (2010) attribution theory and Heckhausen et al.’s (2010) dual-process model in encouraging overly confident social science students to consider both primary control (controllable attributions) and secondary control strategies (positive reappraisal; Hall et al., 2006c). In addition to overconfident students in the intervention condition obtaining 10% higher final course grades relative to controls, their expectations were also lower (more realistic) 5 months later as compared to overconfident control participants whose grades were, on average, 17% lower than expected. Thus, whereas previous research based on Heckhausen et al.’s (2010) life-span theory shows clear psychological and achievement benefits of discouraging a focus on fixed abilities and encouraging realistic expectations specifically for overly optimistic students, no research to date has evaluated intervention programs for overconfident students in STEM programs addressing the benefits of realistic aspirations for well-being and academic success.

To address this research gap, the present study represented an exploratory evaluation of a brief motivational intervention informing students in STEM degree programs of the potential psychological and achievement benefits of realistic expectations following academic setbacks. In line with Heckhausen et al. (2010), Hypothesis 1 proposed that students in the intervention condition would report more adaptive (slightly lower) aspirations with respect to their academic success as compared to controls that were expected to report notably high expectations (cf. Hall et al., 2006c). Hypothesis 2 further proposed that the intervention would lead to better psychological adjustment relative to controls. Finally, Hypothesis 3 proposed that participation in the intervention condition should lead to higher long-term academic achievement relative to controls following from anticipated gains in motivation due to attainable aspirations.

## Materials and Methods

### Participants

Fifty-two undergraduates enrolled in STEM degree programs and completing introductory-level STEM courses at a North American research-intensive university were recruited in the winter semester for a three-phase study via mass emails from faculty deans and students affairs directors. Participants were primarily enrolled in the biological sciences (84.5%) and were additionally enrolled in the physical sciences (10.3%) as well as engineering and related programs (5.2%). The majority of participants were female (61.5%) and in their first year of study (84.6%) with an average age of 18.25 (*SD* = 0.52). Participants’ ethnic backgrounds included Asian American/Pacific Islander (71.2%), Caucasian (15.4%), African American (3.8%), and others (9.6%), with most reporting English as their first language (79%). Participants’ reported high-school grades showed 89% to have graduated with a GPA of 85% or higher (*M* = 90.20, *SD* = 5.46), with participant attrition found to not significantly differ as a function of gender [χ^2^(1, *N* = 52) = 1.02, *p* = 0.60], age [*F*(1,51) = 0.1.19, *p* = 0.31], ethnicity [χ^2^(1, *N* = 52) = 1.02, *p* = 0.11], English as first language [χ^2^(1, *N* = 52) = 1.35, *p* = 0.51], discipline [χ^2^(1, *N* = 52) = 0.87, *p* = 0.93], high-school grades [*F*(1,45) = 0.91, *p* = 0.41], or intervention condition [χ^2^(1, *N* = 37) = 1.52, *p* = 0.32].

### Intervention Content

The intervention and control conditions consisted of three components, and utilized specific techniques consistent with those commonly employed in motivational intervention research with undergraduates based on Weiner’s attribution theory (i.e., AR exercises; Haynes et al., 2009). First, participants completed a difficult GRE-type aptitude test (Abstract Reasoning and Abilities Test, ARAT) used previously in AR research as a simulated failure experience (for previous findings on the efficacy of the ARAT as a failure priming task, see Hall et al., 2004), after which they were immediately debriefed of its intended use to prime failure-related cognitions. Second, participants were provided a brief handout containing either intervention content (*n* = 20) or a control group reading (*n* = 17) to be reviewed individually. The handout content was derived directly from Heckhausen et al.’s (2010) life-span theory of motivation and related empirical research (e.g., Tomasik et al., 2009; Tomasik and Salmela-Aro, 2012) in briefly outlining the risks of unrealistic aspirations as well as benefits of downgrading expectations in an academic context. The handout contrasted statements such as “Anything less than the best is failure” with more realistic alternatives and supporting rationales (e.g., “Overly high goals can make you feel like a failure even when you succeed”). Participants in the control group completed a similarly formatted reading addressing the science behind medical myths (e.g., “You only use 10 percent of your brain”). Finally, a writing exercise was administered in both the intervention and control conditions that required participants to summarize and discuss the main points of the handout (depth), provide examples of the issues discussed (breadth), explain how they could apply the content in their own lives (personal structure), and share their emotions concerning academic failure (cf. writing interventions, Pennebaker, 1997; elaborative processing, Entwistle, 2000).

### Dependent Measures

Descriptive statistics (ranges, means, and standard deviations) and scale reliability for the academic- and domain-general self-report measures, as well as sessional achievement outcomes obtained from student records, are presented in **Table 1**.

**Table 1:** Descriptive statistics for study measures at time 1/2.

Variable	Observed range	*M*	*SD*	α (*r*)
Expectations: Academic	2-7/1-7	5.29/5.39	1.32/1.26	(0.77/0.63)
Expectations: Achievement	2.35-3.98/2.55-3.95	3.36/3.37	0.39/0.39	
Optimism	6-29/13-29	21.50/21.90	4.74/4.17	0.81/0.86
Achievement motivation				
Mastery	7-27/11-26	20.83/19.55	4.34/3.57	0.81/0.74
Performance	7-28/10-28	24.60/24.52	4.12/3.90	0.77/0.87
Academic emotions				
Enjoyment	10-30/14-27	20.27/19.61	4.73/3.32	0.77/0.55
Anxiety	7-29/6-25	16.75/17.19	5.32/5.04	0.78/0.80
Boredom	6-24/6-23	14.97/15.23	4.78/4.84	0.87/0.86
Illness symptoms	8-36/10-28	17.77/16.35	5.28/5.78	0.72/0.80
Depression	11-31/10-31	21.40/20.90	5.27/5.93	0.76/0.71
GPA				
Winter year 1	0.65-4.00	3.07	0.62	
Spring year 1	1.81-4.00	3.09	0.52	
Fall year 1	1.55-4.00	3.12	0.68	
Winter year 2	0.00-4.00	3.04	0.72	
Spring year 2	1.65-4.00	3.12	0.57	

#### Academic Expectations

Academic expectations were assessed utilizing two measures evaluating both global and specific expectations for academic success. The first measure consisted of a single general academic expectation item: “I expect to do very well overall at university this year” (Likert; 1 = *Very Unsuccessful*, 10 = *Very Successful*). The second measure consisted of two averaged items that more specifically evaluated students’ expected future achievement including “What GPA do you expect to obtain at the end of this semester?” (possible range: 0–4.00) and “What overall GPA do you expect to have by the upcoming fall semester (your total cumulative GPA including all previous semesters)?” [possible range: 0–4.00; inter-item correlation at Time 1/2: *r*(48/31) = 0.77/0.63].

#### Optimism

Six Likert-style items from Scheier and Carver’s (1992) Life Orientation Test (LOT) were summed to provide a domain-general measure of dispositional optimism (e.g., “In uncertain times, I usually expect the best”; 1 = *strongly disagree*, 5 = *strongly agree*; α_T1/T2_ = 0.81/0.86).

#### Achievement Motivation

To assess achievement motivation, two four-item measures of achievement goal orientations were adapted from Pintrich et al. (1989) and included items such as “I prefer course material that really challenges me so I can learn new things” (mastery orientation; α_T1/T2_ = 0.81/0.74) and “If I can, I want to get better grades in my classes than most of the other students” (performance orientation; α_T1/T2_ = 0.77/0.87; 1 = *not at all true of me*, 7 = *very true of me*).

#### Academic Emotions

Three learning-related emotions were assessed using six-item, five-point Likert scales adapted from the Academic Emotions Questionnaire (AEQ; Pekrun et al., 2005). Following a modified preamble asking students to reflect on their experiences in a common core class in which they were enrolled that semester, the item to follow assessed their learning-related enjoyment (α_T1/T2_ = 0.77/0.55; e.g., “I enjoy learning new things”), anxiety (α_T1/T2_ = 0.78/0.80; e.g., “When studying the material in this course, my heart rate increases because I get anxious”), and boredom in that class (α_T1/T2_ = 0.87/0.86; “When studying for this course, I feel bored”; 1 = *not at all true*, 5 = *completely true*).

#### Illness Symptoms

An eight-item symptom checklist derived from the Cohen–Hoberman Inventory of Physical Symptoms (CHIPS; Cohen and Hoberman, 1983) was used to assess how often during the last month (α_T1/T2_ = 0.72/0.80; 1 = *not at all a week* to 5 = *5 or more times a week*) students experienced the following symptoms: sleep problems, headaches, low energy, muscle tension, fatigue, stomach pain, heart pounding, and poor appetite.

#### Depression

Mild depressive symptomology was assessed using the ten-item Center for Epidemiologic Studies Depression scale (CES-D; α_T1/T2_ = 0.76/0.80; Radloff, 1997) in which participants were asked how often (1 = *rarely or none of the time*, 4 = *most or all of the time*) during the last month they felt as described (e.g., “my sleep was restless,” “I felt depressed”).

#### Grade Point Average (GPA)

Six sessional GPAs were obtained from the registrar’s office for the fall semester prior to study recruitment (evaluated as a baseline covariate), the winter, spring, and fall semesters of the calendar year in which participants were recruited, as well as winter and spring semesters of the following year. Each sessional GPA consisted of the mean grade obtained across all courses completed during that semester.

### Procedure

In Phase 1 (January-February), participants completed an online questionnaire including demographic items and the self-report measures (∼15 min). Following the questionnaire, students selected one of two in-person sessions to attend for Phase 2 (April) in which intervention or control activities were completed (sessions were randomly assigned to administer experimental or control protocols; ∼30 min). Approximately 4 months after the in-person Phase 2 session, Phase 3 required students to again complete the online questionnaire including the self-report measures. Students’ sessional GPA and course load were subsequently obtained from registrar’s office for the preceding fall semester, for the winter semester in which they were recruited (Year 1), and for the following four semesters (spring and fall Year 1, winter and spring Year 2). As an incentive for participation, students who completed Phases 1 and 2 of the study were entered into a raﬄe for one of four iPods, with students who completed Phase 3 entered into an additional raﬄe for multiple bookstore gift certificates ranging from $10 to $50.

## Results

One-way analyses of covariance (ANCOVAs) were used to evaluate the effects of the downgrading intervention on self-report variables (Hypotheses 1 and 2), with a repeated-measures ANCOVA conducted to assess treatment effects on long-term achievement (five subsequent sessional GPAs; Hypothesis 3). No significant initial differences (chi-square analyses, *t*-tests, *p* < 0.05) were observed between the experimental conditions on baseline levels of the study outcomes nor potentially confounding background measures (age, gender, English as first language, ethnicity, discipline, year of study). Nevertheless, the covariates evaluated included not only baseline levels of each study outcome assessed (e.g., prior sessional GPA as covariate when assessing effects on post-intervention GPAs) but also participants’ gender as per prior research on gendered overconfidence in STEM domains (e.g., Hyde et al., 2008; Goetz et al., 2013), but also high-school grade and year of study consistent with published motivational intervention research with undergraduates (cf. Haynes et al., 2009). Covariate-adjusted means and standard deviations for the experimental conditions are provided in **Table 2**.

**Table 2:** Means and standard deviations by intervention condition.

	Intervention		Control			
Variable	*M*	*SD*	*M*	*SD*	*F*	$\eta_{p}^{2}$
Expectations: Academic	5.85	0.32	4.71	0.49	6.49^∗^	0.22
Expectations: Achievement	3.42	0.36	3.36	0.40	0.29	0.01
Optimism	22.89	2.53	19.82	5.07	8.05^∗∗^	0.26
Mastery orientation	19.37	3.80	19.81	3.57	0.11	0.01
Performance orientation	23.77	4.67	25.25	2.31	1.99	0.08
Enjoyment	18.95	2.98	20.16	3.32	1.15	0.05
Anxiety	16.15	4.73	18.04	5.56	1.61	0.07
Boredom	15.13	5.02	15.73	4.62	2.14	0.01
Illness symptoms	15.18	6.88	18.25	4.61	1.89	0.08
Depression	20.17	6.56	22.18	5.40	0.87	0.04
GPA (average, five semesters)	2.96	0.45	3.25	0.47	5.38^∗^	0.17

*Time 2 means statistically adjusted for covariates including gender, high-school grade, and year of study. ^∗^*p* < 0.05, ^∗∗^*p* < 0.01*.

Significant treatment effects were observed on students’ general expectations for academic success, *F*(1,23) = 6.49, *p* = 0.018, $\eta_{p}^{2}$ = 0.22, as well as global optimism levels, *F*(1,23) = 8.05, *p* = 0.009, $\eta_{p}^{2}$ = 0.26, with results showing students in the intervention condition to report higher post-intervention academic expectations (*M* = 5.85) and optimism (*M* = 22.89) compared to students in the control condition (*M* = 4.71; *M* = 19.82; respectively). Although the results for anxiety and performance goal orientations were in the expected directions, one-way ANCOVAs showed no significant treatment effects on the remaining self-report outcome measures. Finally, a repeated measures ANCOVA revealed a significant between-subjects, omnibus treatment effect on GPA, *F*(1,27) = 5.38, *p* = 0.028, $\eta_{p}^{2}$ = 0.17. However, the direction of this effect was contrary to that hypothesized, with participants in the intervention condition (*M* = 2.96) obtaining consistently *lower* GPAs over the subsequent 2-year period relative to controls (*M* = 3.25).

## Discussion

### Hypothesis 1: Academic Expectations and Optimism

According to the first hypothesis, participants in the intervention condition were expected to demonstrate better adjusted (i.e., slightly lower) aspirations as part of becoming better calibrated with their highly achievement-oriented academic reality and potential for academic disappointment (e.g., non-admission into a medical program). These results instead showed students in the intervention condition to report *higher* levels of optimism as well as general expectations for academic success relative to controls, a finding that although is positive, is contrary to that expected following our intervention addressing the downgrading of aspirations. One possible explanation is that when overconfident students who are highly ego-involved in a domain encounter failure, they often engage in self-enhancement to maintain their self-esteem (Robins and Beer, 2001). In other words, reminding these students of possible setbacks in an academic program in which they were heavily invested may have triggered a defensive overcompensation in expectation levels. As students in STEM programs are likely to have their self-esteem closely tied to their performance (Perez, 2012), it is therefore possible that the intervention was perceived not as informative but rather a threat to one’s self-concept as a successful student. Alternatively, it is possible that whereas lower aspirations may have occurred immediately following the intervention, greater optimism and expected success 4 months later may indeed reflect genuine, longer-term motivational gains assumed to follow from encouraging students to consider the importance of downgrading following setbacks (Hypothesis 2). Given a similar pattern of encouraging findings on the other motivational and adjustment measures, these findings may reflect the motivating as opposed to threatening nature of the intervention program.

### Hypothesis 2: Achievement Motivation and Well-Being

Following from Heckhausen et al.’s (2010) life-span theory of motivation, the second study hypothesis proposed that sustained motivational resources resulting from a greater appreciation of the role of realistic expectations in challenging academic settings would contribute to greater psychological and physical well-being and more adaptive achievement goals. Although an encouraging pattern of results was observed showing intervention participants to report higher levels of enjoyment and mastery goal orientation, as well as lower anxiety and boredom relative to controls, these treatment effects did not reach significance. As such, our findings did not provide clear support for Hypothesis 2 and are not in line with previous studies showing significant motivational and well-being benefits from downgrading as a motivational strategy (e.g., Wrosch et al., 2000; Heckhausen and Tomasik, 2002; Tomasik et al., 2009) or related research on the drawbacks of pursuing unrealistic goals in young adulthood (Wrosch et al., 2003, 2007). Instead, these findings suggest that whereas the expectancy variables directly targeted by the intervention were significantly impacted over a 4-month period, similar changes on psychological outcomes not directly addressed in the intervention were not found. However, although it is possible that the narrow program focus may have limited the range of benefits observed, the lag between the intervention and follow-up questionnaire was shorter than in typical AR studies (e.g., 6 months; Hall et al., 2006c) preventing the detection of effects that require more time to emerge (e.g., for students to experience some academic setback and meaningfully apply downgrading strategies).

### Hypothesis 3: Academic Performance

Due to the highly demanding and competitive nature of STEM degree programs, academic setbacks and disappointment represent an unfortunate reality for many students in these disciplines. According to Hypothesis 3, participants in the intervention condition were expected to gain a greater appreciation of how realistic aspirations can help students preserve their motivational resources in the face of academic difficulties, leading to sustained personal well-being and higher GPAs over time relative to controls. The present findings revealed that in direct contrast to this hypothesis, students in the intervention condition demonstrated consistently lower grades following the intervention in comparison to students in the control condition who received no intervention. Given the large magnitude of the treatment effect (>0.14, Cohen, 1969), and that it was observed controlling for not only baseline GPA (from the preceding term) but also students’ course load and demographic background variables (age, gender), we can reliably attribute the poorer performance observed to the intervention content addressing the downgrading of academic aspirations as opposed to potential critical confounds.

As noted previously, one explanation for this discouraging result may involve the possibly ego-threatening nature of the failure-oriented intervention for STEM students (Robins and Beer, 2001; Perez, 2012) who may have reacted defensively, overcompensated with higher expectations, and pursued even more challenging goals thereby increasing their chances of failure and disengagement (e.g., self-handicapping). This interpretation is consistent with studies showing students with particularly high self-esteem to experience lower grades (Hall et al., 2010) and job interview success (Hall et al., 2011) following otherwise effective interventions encouraging persistence, likely due to threated self-perceptions as high-ability students (vs. high-effort students). However, given slightly greater well-being and motivation for intervention participants, and a lack of higher negative affect, it is also plausible that the intervention prompted STEM students to demand less of themselves with respect to their persistence, leading to lower yet stable performance (vs. declines indicating disengagement).

This alternative explanation is consistent with studies showing self-esteem enhancement programs to negatively affect both students’ academic self-concept (Wade et al., 2003) and achievement (Forsyth et al., 2007). Whereas these self-esteem bolstering initiatives encouraged struggling students to view themselves in a more positive light, as a corollary, there were also likely encouraged to implicitly perceive their present poor performance as an acceptable standard thereby decreasing their motivation for personal improvement. It is therefore possible that the present intervention in which STEM students were explicitly encouraged to consider the importance of adopting more attainable academic goals may have resulted in downgraded aspirations (e.g., shortly following the program), greater hope of achieving these more attainable standards (4 months later), as well as lower persistence and performance levels. However, as short-term changes in expectancy were not assessed, indicators of persistence were not examined, and no clear emotional benefits of participating in the downgrading intervention were observed beyond greater optimism and expectations, further research is required to more substantively evaluate this hypothesis.

### Limitations and Future Directions

First, it should be noted that although the present study is consistent with emerging findings highlighting academic overconfidence in post-secondary STEM programs (e.g., Reuben et al., 2013; Bench et al., 2015), study participants were not prescreened with respect to overconfidence levels (e.g., low grades combined with high expectations; cf. Haynes et al., 2006). Thus it is possible that our recruitment protocols advertising the study as examining motivation topics may not have attracted already motivated and confident students, as suggested by the majority of study participants being female (61%) who are commonly found to report lower self-efficacy and performance expectations relative to males in mathematics and science domains (e.g., Hyde et al., 2008; Goetz et al., 2013). As such, future larger-scale research with STEM students in which baseline overconfidence is clearly demonstrated, baseline levels are examined as moderating variables (e.g., low/moderate/high), or at-risk students are exclusively preselected for participation is warranted to more specifically examine the potential benefits and risks of the present intervention for overly confident students in STEM programs.

Second, as the limited sample size of this exploratory study may have contributed to a lack of significant findings for some measures (e.g., well-being, goal orientation), research with larger samples is needed to replicate the present findings. Based on power analyses conducted with G^∗^Power software (Faul et al., 2009), a sample of at least 58 participants is recommended to ensure 80% power to detect equivalent effects (*p* < 0.05; i.e., the between-subjects ANCOVA effect on achievement). Third, as the present pilot study employed a quasi-experimental design in that intervention conditions were randomly applied to experimental sessions (vs. participants), future research using true random assignment is recommended to replicate our results. Fourth, although this study evaluated multiple indicators of psychological adjustment, motivation, and achievement, it did not assess measures of self-esteem and persistence (e.g., hours worked, assistance sought), nor did it directly evaluate students’ perceived self-regulatory use of downgrading strategies (e.g., post-intervention manipulation check). Accordingly, longitudinal research with multiple short- and long-term follow-ups in which these potential and hypothesized mediators are also evaluated is recommended to better determine whether STEM students reacted defensively to the program or opted to preserve their well-being by reducing unmitigated persistence at the expense of their grades.

Finally, given that the narrow intervention focus on downgrading unrealistic expectations neglected to mention other motivational variables consistently found to predict achievement in college students (e.g., perceived competence, utility value; see Robbins et al., 2009), future studies in which downgrading content is combined or contrasted with more traditional motivational messages (e.g., personally controllable attributions for failure experiences; Hall et al., 2007) or higher-order motivational constructs (e.g., purposeful enactment of realistic action plans; Han, 2015) are encouraged to provide a more balanced motivational perspective and possibly mitigate achievement risks. In sum, the present exploratory findings underscore the importance of further research on how to better encourage students in STEM disciplines to consider the psychological risks of unrealistic expectations as well as the potential benefits of downgrading aspirations following setbacks encountered in these challenging degree programs.

## Author Contributions

NH conducted data collection, statistical analysis, and manuscript writing. AS conducted statistical analysis and manuscript writing.

## Conflict of Interest Statement

The authors declare that the research was conducted in the absence of any commercial or financial relationships that could be construed as a potential conflict of interest.
